# Safety assessment of (S)-Equol: Subchronic toxicity study in Sprague Dawley Rats

**DOI:** 10.1016/j.toxrep.2024.101823

**Published:** 2024-11-19

**Authors:** Seethakallu Ramachandraiah AnandaKumar, Mukund Handral, Srinivas Seekallu

**Affiliations:** a Department of Pharmacology, PESU Institute of Pharmacy, PES University, Bangalore, Karnataka-560100, India; b Department of Pre-Clinical Research, Anthem Biosciences Pvt. Ltd., #49, F1 & F2, Canara Bank Road, Bommasandra Industrial Area Phase 1, Bommasandra, Bengaluru, Karnataka-560099, India

**Keywords:** (S)-Equol, Subchronic toxicity, Enantiopure, Nutraceutical, No Observed Adverse Effect Level

## Abstract

(S)-Equol is a chemically synthesized nutraceutical compound and its consumption provides several health benefits for humans. The new nutraceutical, enantiopure (S)-Equol was studied for acute and sub-chronic toxicity in Sprague Dawley Rats. The oral acute toxicity study showed that (S)-Equol is safe > 2000–5000 mg/kg body weight and it classified into GHS category 5/Unclassified. The repeated dose administration of (S)-Equol at dose levels of 20, 60, and 160 mg/kg body weight for 14 days and 250, 500, and 1000 mg/kg body weight for 90-consecitve days. The 14 days repeated-dose toxicity study showed no adverse effects in Sprague Dawley rats. The 90-day repeated dose toxicity study showed a reduction in body weight gain than that of control group. No treatment-related contrary effects were perceived on haematology, clinical chemistry, coagulation, urine parameters analysed, organ weights (absolute and relative), neurological and ophthalmological examination. No treatment-related abnormal gross pathological findings were obtained from gross necropsy. However, as a treatment-related effect, a significant decrease in cholesterol levels for 14 and 90 days of repeated dose administration was observed, which is considered as a pharmacological class effect of the (S)-Equol. In comparison to the corresponding vehicle control group, the high dose treatment group for both sexes showed no treatment-related histopathological abnormalities. For female rats, the no-observed-adverse-effect-level (NOAEL) was 250 mg/kg/day and for male rats, the lowest-observed-adverse-effect level (LOAEL) was 250 mg/kg/day.

## Highlights

• Decrease in total cholesterol was pharmacological class effect of (S)-Equol.
• (S)-Equol does not affect the blood chemistry and hematology parameters of Rats.
• (S)-Equol does not alter histopathological microscopic changes of organs in Rats.
• Functional observational battery (FOB) was normal in male and female Rats
• No-observed-adverse-effect level (NOAEL) for female rats was 250 mg/kg/day.
• Lowest-observed-adverse-effect level (LOAEL) for male rats was 250 mg/kg/day.

## Graphical Abstract

**Figure:**
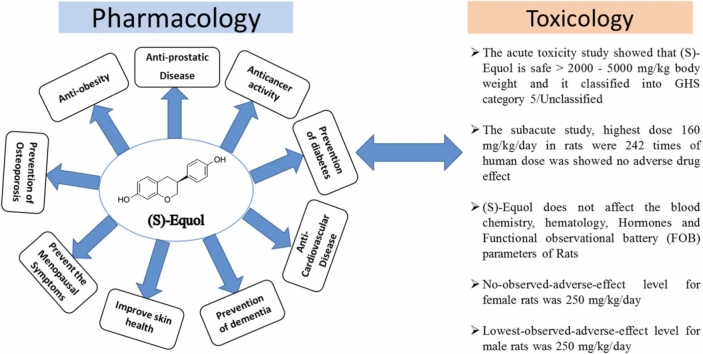

## 1 Introduction

The estrogenic action of soy isoflavonoids such as daidzein and genistein have fascinated several researchers owing to their promising activity in cancer treatment and to treat menopausal risks [4]. Though less active than hormone, soy isoflavones are phytoestrogens that resemble 17-β-estradiol and have estrogen-like activity. An isoflavone-derived metabolite called Equol is created by microbes in the small intestine's distal region from daidzein [22]. Every individual differ ability to produce equol. The 30–50 % of the population, have the gut bacteria required to turn daidzein into equol [14]. Genetics, dietary habits, and gut flora make a difference in one's capacity to produce equol. A invention established by a Japanese company Otsuka Pharmaceutical Co., Ltd., termed as SE5-OH contained a prominent composition of equol. The product was obtained from the soy fermentation using *Lactococcus garvieae* bacteria [28]. Most of the reported animal models to study the equol uses racemic equol (± equol) [11], [3] as it is commercially available.

Equol is a non-polar phenolic isoflavane with a chemical formula 4’, 7-isoflavandiol. The compound has asymmetric carbon at C3 position and results in racemic mixture with *R*(-) and *S*(-) forms. Nevertheless, *S*(-)-Equol is detected to be converted by bacterial conversion of daidzein [20], [9] and both the enantiomers present varied biological activity. (*R*)-form and (*S*)-form are known to possess greater activity towards ERα (estrogen receptor α) and ERβ (estrogen receptor β) [16]. Therefore, research employing enantiopure material is important to clarify the specific biological consequences of such metabolites.

As discussed above, the (S)-Equol can be obtained by daidzein, the racemate was synthesized and separated by chiral HPLC [16] and bacterial conversion of dihydrodaidzein [27], [26]. Xiu-Ling et al., synthesized *S*-Equol using newly isolated bacteria from human feces [27]. The bacterium was not able to synthesize (S)-Equol from daidzein, dehydroequobl and tetrahydrodaidzein, but synthesized using dihydrodaidzein. (S)-Equol has been successfully produced *in vitro* utilizing a variety of microorganisms [30]. Uchiyama et al., for instance, were able to identify *Lactococcus* 20–92, a bacteria which could produce as (S)-Equol from human feces and successfully produce (S)-Equol from soy germ that was rich in daidzein [23]. It seems that these particular Equol-producing bacteria [12], [17], rather than genes, determine an individual's (S)-Equol generating status in the intestine [25], [5]. Furthermore, the state of (S)-Equol produced is very stable [7]. Though several synthetic methods are available for (S)-Equol, the acute and subchronic studies for its administration and NOAEL of the same is yet to be discovered. In the present work, 100 % pure enantiomer (S)-Equol was collected from chemistry department of Anthem Biosciences. The (S)-Equol was assessed for acute, subacute and subchronic studies using Sprague Dawley Rats. A 14 and 90 days repeated dose studies were conducted with the administration of (S)-Equol of varied concentrations to animals of four groups of each sex. The investigation was further extended to subchronic toxicity study under which the changes in body weight, feed consumption, haematology, neurological changes, vaginal cytology, organ weights and blood chemistry, and pathological studies were examined to understand the toxicity of the considered enantiopure (S)-Equol.

## 2 Materials and methods

### 2.1 Test item

A characterised and an enantiopure (S)-Equol was procured from the Department of Chemistry, Anthem Biosciences Pvt. Ltd., Bengaluru, India with the Lot no. A012000810 and A022000370. (S)-Equol was a dry, solid, half-white powder with 100 % purity measured with HPLC (% w/w). It was stored in a dry, clean, and well-ventilated area at room temperature (RT) (19–25 °C). The prepared (S)-Equol formulation was stored refrigerator under 2–8 °C temperature, used within seven days for the dose administration of animals.

### 2.2 Animals

The present study used SPF Sprague Dawley [CD(SD)IGS] rats procured from Hylasco Biotechnology Pvt. Ltd., Hyderabad, and housed in Anthem Biosciences Pvt. Ltd. Bangalore, India, an AAALAC approved animal facility. The multiple ages animals was procured for toxicity studies. The rats were acclimatized for 5 days in all studies and were of ∼9 weeks in acute, 7–8 weeks in 14 days repeated dose toxicity study and 5–6 weeks in subchronic toxicity study at the start of treatment. A maximum of 2 animals were accommodated in each typical polycarbonate cage and maintained at a standard laboratory conditions, 12 h light/12 h dark cycle, 30–70 % of relative humidity, and the temperature of 22 ± 3 °C. The animals were given ad libitum water and rat maintenance gamma irradiated feed of batch No. 202007231845 and 202009242016 (Manufactured by Altromin Spezialfutter GmbH & Co. KG). Protocol of the study was obtained an approval with a number ABD/IAEC/PR/256-21–24 by Institutional Animal Ethics Committee (IAEC).

### 2.3 Acute oral toxicity study

An acute oral toxicity study was carried out using female SD Rats. Overnight fasted animals were administered with (S)-Equol at 300 mg/kg body weight, and since no impermanence or adverse clinical signs of toxicity were observed, the next higher dose of 2000 mg/kg body weight was selected. Overnight fasted animals were administered with (S)-Equol at 2000 mg/kg body weight. On 15^th^ day, study animals were euthanized using CO_2_ overdose and the gross necropsy was conducted. The study was conducted according to OECD 423 test guideline Acute Oral Toxicity – Acute Toxic Class Method, Annex 2 c: Starting dose is: 300 mg/kg bw, Adopted: 17th December 2001 [19].

### 2.4 Dose Range Finding Study

Twenty males and twenty females of approximately seven to eight week old Sprague Dawley rats were assigned into four groups of five animals per each group ^(5/sex/group) considered for 14 days toxicity study^. Dose levels of 0 (vehicle control, VC), 20 (low), 60 (mid) and 160 (high) mg/kg body wt. were selected. The formulation of the (S)-Equol was prepared just before administration using 100 % (v/v) of 1 % (w/v) HPMC in ultrapure water as vehicle. Every animal was seen once a day for clinical symptoms, twice a day for mortality and morbidity, and once a week for a thorough clinical assessment, body weight, and feed intake. Day 15 saw the analysis of blood and collected plasma for for haematology and clinical chemistry parameters respectively. The animals were euthanized with CO_2_ asphyxiation followed by exsanguination and given a thorough pathological investigation. For histological analysis, the designated organs were gathered, weighed, and kept in an appropriate fixative.

### 2.5 Sub-chronic toxicity studies

#### 2.5.1 Experimental design

According to body weight stratification and randomization four groups with 10 animals in each group (10/sex/group) were considered for the study. The first group G1 is VC, received daily once 1 % (w/v) HPMC (hydroxypropyl methylcellulose) alone orally, group G2, G3 and G4 received test item of 250 (low), 500 (mid) and 1000 (high) doses mg/kg/day at a dose volume of 5 mL/kg/day for 90 consecutive days. Every animal was checked twice a day for death and illness and once a day for clinical indicators of toxicity. The body masses and feed intakes were noted every 7 days and the group mean (g/animal/day/week) was figured. The study was conducted according to OECD 408, Adopted: 25th June 2018 - Repeated Dose 90-Day Oral Toxicity Study in Rodents [18].

#### 2.5.2 Homogeneity of (S)-Equol formulation

On days 0 and 78 of the treatment period, the homogeneity (3 layers, top, middle, and bottom) and dose confirmation analysis of the (S)-Equol formulations at concentrations of 50, 100, and 200 mg/mL were evaluated using a validated HPLC method in accordance with a method validation study. To rule out any possibility of contamination of the (S)-Equol, in 1 % (w/v) HPMC samples used as a vehicle were examined.

#### 2.5.3 Neurological and ophthalmological examination

The 13^th^ week was dedicated to the neurological and functional evaluation of the animals in the high dosage (G4) and VC (G1) groups. Numerous neurological examinations were conducted, including the home cage examination such as posture, vocalization, respiratory pattern, palpebral closure, the handheld examination like ease of removal and handling, salivation, hair coat, lacrimation, muscle tone chromodacryorrhea, the open field examination such as number of urinations, piloerection, gait, arousal, clonic or tonic involuntary movement, mobility, stereotype, exophthalmos, number of defecations and rearing, the neuromuscular examination like hind limb foot splay, forelimb grip strength test and physiological measurements. Animals from vehicle control and high dose treated were examined for eye to verify the (S)-Equol effect on repeated dose administration using indirect ophthalmoscope.

#### 2.5.4 Hematology and serum biochemistry

All the animals were anesthetised by isoflurane on the 91 day, and blood samples were collected from retro-orbital route in to tubes with K_2_EDTA and lithium heparin for the haematology and clinical chemistry parameters respectively. The Siemens Dimension Xpand Plus clinical chemistry analyzer was used to analyze the clinical chemistry parameters and the ADVIA 2120 was used to analyze the hematological parameters. The KC4 Delta Tcoag coagulation analyzer was used to measure the prothrombin and activated partial thromboplastin time, while the Siemens Rapidchem RC744 electrolyte analyzer was used to estimate the electrolytes.

#### 2.5.5 Urinalysis

On 13^th^ week of treatment to collect the urine, animals kept in metabolic cages were fasted overnight and samples were collected. The collected urine was subjected for microscopic examination. The Clinitek Status+ urine analyzer instrument was used to measure urine biochemical parameters.

#### 2.5.6 Morphology studies

On day 90, when the treatment came to an end, the animals were fasted for the overnight, their fasted body weights were recorded, and blood samples were collected. After blood collection, all the animals were euthanized by CO_2_ overdose. detailed gross necropsy including careful examination of the external surface of the body, all orifices, cranial, thoracic and abdominal cavities and their contents were performed.

#### 2.5.7 Histopathological examination

All organs specified by OECD 408 were collected and tissues were subjected for fixation followed by trimmed, processed, embedded in paraplast to prepare tissues blocks. The prepared tissue blocks were sectioned and stained with haematoxylin and eosin. All the prepared slides were observed for microscopic changes in tissues. The tissues on the designated list, which included all macroscopically aberrant tissues from all animals in the high dosage group (G4) and vehicle control (G1), were subjected to the histological analysis by veterinary pathologist. Following treatment-related lesions in high dose group, the low-dose (G2) and mid-dose (G3) study groups had their target organs examined as well.

#### 2.5.8 Statistical analysis

All the results were statistically analysed using Graph Pad Prism. A one-way ANOVA followed by student't t test unpaired two tailed was performed where only two groups were used for comparison and Dunnett's post-test was performed. All the statistical analysis was assessed with a 95 % for p<0.05, 99 % for p<0.01, 99.9 % for 0.001 level of confidence, statistically significant changes obtained was designated by the superscripts throughout the manuscript as * (p<0.05), ** (p<0.01), and *** (p<0.001).

## 3 Results

### 3.1 Acute toxicity

No abnormal clinical signs and mortality were observed. The investigation of acute toxicity study revealed the single oral dose of (S)-Equol is tolerable at > 2000–5000 mg/kg body weight in female Sprague Dawley rats. The (S)-Equol can be classified to Globally Harmonized System (GHS) category 5/Unclassified for classification of chemicals and mixtures.

### 3.2 Dose Range Finding study

The 14 days repeated oral gavage of (S)-Equol at 20 (low), 60 (mid) and 160 (high) mg/kg body weight showed no contrary clinical signs, mortality/morbidity, or effects on body weight, feed intake, haematology, clinical chemistry, coagulation parameters, or organ weights. On microscopic examination, no treatment-related adverse histopathological findings were observed in the high dose group 160 mg/kg/day of both the genders.

### 3.3 Sub-chronic toxicity

#### 3.3.1 Formulation Homogeneity and Concentration Confirmation

In sub-chronic study, dose formulations of (S)-Equol prepared in vehicle at concentrations of 50, 100 and 200 mg/mL on day 1 (pre-dose) and week 13 of the study were analysed for homogeneity and the results were within acceptable limits (Limits: % Recovery: 85 to 115 % and % RSD: ≤ 10) Table 1.

**Table 1:** – Formulation concentration confirmation.

Homogeneity of (S)-Equol formulation on Day 0							
**Layer**		50 mg/mL		100 mg/mL		200 mg/mL	
		Mean Measured Content of (S)-Equol (µg/mL)	% Mean Recovery of the Claimed Concentration	Mean Measured Content of (S)-Equol (µg/mL)	% Mean Recovery of the Claimed Concentration	Mean Measured Content of (S)-Equol (µg/mL)	% Mean Recovery of the Claimed Concentration
TOP	Mean	58.47	97.45	58.67	97.79	58.84	98.07
	SD	0.503	0.838	0.053	0.084	0.017	0.029
	%CV	0.86	0.86	0.09	0.09	0.03	0.03
MID	Mean	58.67	97.79	58.69	97.82	58.71	97.85
	SD	0.021	0.032	0.024	0.04	0.024	0.04
	%CV	0.04	0.03	0.04	0.04	0.04	0.04
Bottom	Mean	58.71	97.85	58.77	97.95	58.73	97.89
	SD	0.031	0.052	0.099	0.169	0.04	0.067
	%CV	0.05	0.05	0.17	0.17	0.07	0.07
Homogeneity of (S)-Equol formulation on Day 78							
TOP	Mean	64.29	107.15	64.22	107.03	63.25	105.42
	SD	0.01	0.017	0.021	0.036	0.012	0.021
	%CV	0.02	0.02	0.03	0.03	0.02	0.02
MID	Mean	64.98	108.29	63.46	105.77	63.07	105.12
	SD	0.029	0.051	0.073	0.123	0.01	0.02
	%CV	0.04	0.05	0.12	0.12	0.02	0.02
Bottom	Mean	64.79	107.98	63.37	105.61	63.23	105.39
	SD	0.028	0.049	0.017	0.025	0.043	0.072
	%CV	0.04	0.05	0.03	0.02	0.07	0.07

Nominal Content (S)-Equol subjected for dose confirmation was 60 µg/mL. Dilution factor is 833.33, 1666.67 and 3333.33 applied for 50, 100 and 200 mg/mL formulation concentration respectively. All figure facts are in mean ± SD and %CV, n=3, n: no of injections for analysis

#### 3.3.2 Clinical signs and mortality

All the animals were planned sacrifice, given the vehicle and (S)-Equol formulation did not exhibit any unfavorable clinical symptoms of toxicity or death/illness.

#### 3.3.3 Body mass and feed intake

**Fig. 1:**
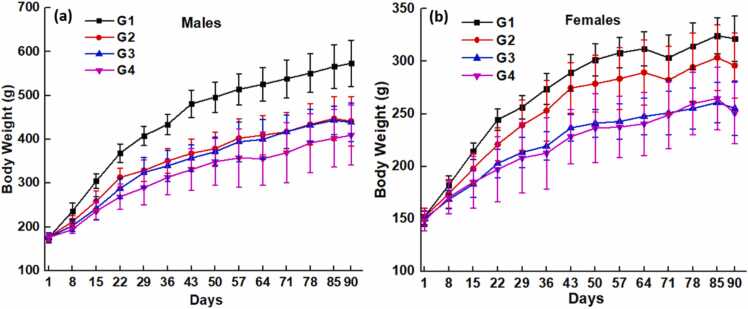
a) Body weights of male rates b) Body weights of female rats for all the treatment groups for 90 days.

The body masses measured for all the four considered groups for 90 days are presented in Fig. 1a (males) and Fig. 1b (females). There was a dose dependent reduction in body mass gain for animals dosed with (S)-Equol compared to the control rats. However, there was a gradual increase in the body weights was observed compared to day 1 body weight.. The increasing body weight was not comparable to vehicle control animals. In male rats, for groups G2 and G3, the difference in the weight gain was not significant, however for G4, treated with 1000 mg/kg of (S)-Equol, the body weight gain decreased than the other two groups treated with 250 and 500 mg/kg of (S)-Equol. Nevertheless, in female rats, groups G3 and G4 fell in the same line while all the groups followed the decreased weight gain trend compared to G1.

Significant reduction in body weight gain, >90, 77 and 73 % in high, mid and low dose males respectively was observed at the end of study (results not shown). Similarly, body weight gain reduction was observed >17 % in low dose and >40 % in mid and high dose females when compare to G1 at the end of study period. The decrease in body weight and weight gain was lesser in females compared to male SD rats. As no adverse effects of the (S)-Equol are observed among the parameters evaluated, the change in body masses are expected to be reversible upon withdrawal of the (S)-Equol treatment with a sufficient recovery period.

The feed consumption measured for both male as well as female groups are presented in Table 2, Table 3. In comparison to G1, the feed consumption was less for the treatment groups. There was also a statically significant reduction for the groups with increased treatment dosage of (S)-Equol which is incidental with decreased body weight, this could be due to lesser body weight gain.

**Table 2:** Effect of 90 days repeated dose administration of (S)-Equol on feed consumption in male Sprague Dawley rats

**Average Feed Consumption - Males**													
**Dose** **(mg/kg/day)**	**Week 1**	**Week 2**	**Week 3**	**Week 4**	**Week 5**	**Week 6**	**Week 7**	**Week 8**	**Week 9**	**Week 10**	**Week 11**	**Week 12**	**Week 13**
**VC**	24.34 ± 0.81	29.32 ± 2.08	30.89 ± 1.52	30.60 ± 1.87	31.80 ± 2.23	32.73 ± 2.12	32.52 ± 2.47	32.48 ± 1.73	30.77 ± 2.37	29.96 ± 3.18	31.02 ± 2.27	31.65 ± 1.18	19.95 ± 2.25
**250**	23.99 ± 1.67	29.04 ± 5.08	29.79 ± 2.22	28.96 ± 2.79	30.03 ± 2.57	29.56 ± 3.50	31.04 ± 2.48	27.34 ± 0.65*↓	27.50 ± 3.24	26.56 ± 1.49	28.95 ± 2.55	27.54 ± 1.62*↓	21.23 ± 2.07
**500**	22.45 ± 1.17*↓	25.46 ± 2.19	26.00 ± 2.23^**^↓	26.62 ± 1.95*↓	26.72 ± 0.92^**^↓	27.15 ± 1.21^**^↓	29.55 ± 3.24	27.27 ± 1.58^**^↓	28.05 ± 2.79	27.54 ± 1.66	28.45 ± 3.02	27.50 ± 1.91*↓	21.94 ± 2.66
**1000**	19.98 ± 0.76^***^↓	21.84 ± 1.92*↓	22.13 ± 1.94^***^↓	23.63 ± 1.20^***^↓	25.33 ± 1.81^***^↓	24.39 ± 1.49^***^↓	27.83 ± 1.69*↓	26.39 ± 4.15^**^↓	27.09 ± 2.51	26.82 ± 2.44	28.18 ± 3.92	24.55 ± 3.39^***^↓	19.99 ± 3.15

All figure facts are in mean ± SD, *P<0.05, **P<0.01 and ***P<0.001 when compared to VC (Vehicle control). ↓-Decrease, ↑-Increase.

**Table 3:** Effect of 90 days repeated dose administration of (S)-Equol on feed consumption in female Sprague Dawley rats.

**Average Feed Consumption - Females**													
**Dose** **(mg/kg/day)**	**Week 1**	**Week 2**	**Week 3**	**Week 4**	**Week 5**	**Week 6**	**Week 7**	**Week 8**	**Week 9**	**Week 10**	**Week 11**	**Week 12**	**Week 13**
**VC**	18.90 ± 0.66	19.89 ± 0.78	21.07 ± 0.58	21.21 ± 0.91	20.65 ± 0.44	21.91 ± 0.61	21.84 ± 0.6	21.68 ± 0.46	20.01 ± 1.37	22.23 ± 2.31	19.48 ± 0.77	19.70 ± 2.50	14.91 ± 0.87
**250**	17.83 ± 2.14	17.68 ± 1.43	18.64 ± 0.76	20.59 ± 1.96	19.87 ± 1.84	21.22 ± 1.74	22.58 ± 0.93	21.07 ± 2.81	21.86 ± 1.94	21.29 ± 2.99	20.67 ± 2.06	22.97 ± 1.46	14.60 ± 1.69
**500**	16.01 ± 0.85^**^↓	15.71 ± 1.92^**^↓	19.51 ± 3.56	18.32 ± 2.63	19.35 ± 2.45	18.25 ± 1.94*↓	18.29 ± 1.26^***^↓	17.00 ± 1.49^**^↓	17.75 ± 2.58	19.95 ± 5.63	18.92 ± 6.09	17.06 ± 2.77	14.56 ± 5.24
**1000**	16.60 ± 0.78^**^↓	17.00 ± 2.87	16.81 ± 2.83*↓	18.81 ± 3.78	18.43 ± 3.39	18.33 ± 3.20*↓	18.49 ± 1.59^***^↓	17.40 ± 2.37*↓	19.38 ± 1.56	20.01 ± 4.72	18.65 ± 2.43	18.17 ± 3.43	14.12 ± 1.76

All figure facts are in mean ± SD, *P<0.05, **P<0.01 and ***P<0.001 when compared to VC (Vehicle control). ↓-Decrease, ↑-Increase.

#### 3.3.4 Neurological investigations

It was observed that until the planned sacrifice, animals given the vehicle and (S)-Equol formulation did not exhibit any unfavorable clinical symptoms of toxicity or mortality/morbidity. However, significant statistical increase in defecation were observed in animals treated with 1000 mg/kg b.w male animals when compared to vehicle control group animals. Other investigated factors turned out to be normal, and the modifications that were noticed were limited to single sex.

#### 3.3.5 Vaginal cytology

On day 90, the estrus cycle stage of every female rat was determined, and no unfavourable effects associated with the treatment were noted.

#### 3.3.6 Urinalysis

When comparing the treated groups of both sexes to the corresponding VC group, no treatment-related adverse effects were seen in the group mean values or in the occurrences of semi-quantitative observations. Females showed an increase in specific gravity, and the other parameters that were assessed turned out to be normal. Thus, the alterations that have been noticed are regarded as accidental.

#### 3.3.7 Hematology and blood chemistry

Compound related decrease in total cholesterol, HDL levels in both the sexes and LDL in only males were observed in present study. The results of hematology and blood chemistry measuring several parameters such as WBC, RBC, HGB, HCT, MCV, MCH, and many were measured and tabulated in Table 4, Table 5, Table 6, Table 7 for male and female rats. Furthermore, significant increase in ALP and GGT in both the sexes and total protein and albumin only in males were also observed when compared with concurrent control. In spite of significant decrease in total cholesterol and high density lipoprotein concentration and increase in ALP and GGT in all the test item treated animals, no significant changes were noticed in liver on histopathological evaluation.

**Table 4:** Effect of 90 days repeated dose administration of (S)-Equol on total blood count in male Sprague Dawley rats.

**Hematology - Males**				
**Parameters**	**Vehicle control**	**250 mg/kg/day**	**500 mg/kg/day**	**100 mg/kg/day**
**WBC (10**^**3**^**cells/µL)**	9.80 ± 2.21	6.91 ± 1.00^**^↓	8.05 ± 2.21	7.60 ± 1.99*↓
**RBC (10**^**6**^**cells/µL)**	9.22 ± 0.30	8.65 ± 0.49*↓	8.61 ± 0.57*↓	8.40 ± 0.59^**^↓
**HGB (g/dL)**	15.93 ± 0.55	15.34 ± 0.73	14.84 ± 0.43^**^↓	14.75 ± 0.52^**^↓
**HCT (%)**	55.15 ± 2.47	54.84 ± 3.62	52.22 ± 2.62	51.09 ± 2.33^**^↓
**MCV (fL)**	59.82 ± 2.22	63.42 ± 2.77	60.70 ± 1.54	60.90 ± 1.67
**MCH (pg)**	17.30 ± 0.44	17.73 ± 0.44	17.28 ± 0.73	17.58 ± 0.64
**MCHC (g/dL)**	28.93 ± 0.80	28.03 ± 0.99*↓	28.47 ± 0.71	28.87 ± 0.46
**Neu (10**^**3**^**cells/µL)**	1.22 ± 0.22	1.06 ± 0.30	1.29 ± 0.40	1.92 ± 1.33
**Lymph (10**^**3**^**cells/µL)**	8.10 ± 1.96	5.56 ± 0.74^**^↓	6.34 ± 1.84*↓	5.32 ± 1.19^***^↓
**Mono (10**^**3**^**cells/µL)**	0.26 ± 0.11	0.19 ± 0.07	0.22 ± 0.11	0.23 ± 0.11
**Eos (10**^**3**^**cells/µL)**	0.13 ± 0.05	0.07 ± 0.03*↓	0.14 ± 0.08	0.09 ± 0.05
**Baso (10**^**3**^**cells/µL)**	0.01 ± 0.01	0.01 ± 0.01	0.01 ± 0.01	0.01 ± 0.00
**Retic (10**^**3**^**cells/µL)**	168.28 ± 26.06	206.56 ± 67.18	201.05 ± 34.64	196.90 ± 54.93
**Platelet (10**^**3**^**cells/µL)**	734.30 ± 146.21	780.70 ± 158.45	842.10 ± 80.66	874.40 ± 66.99*↑
**PT (Sec)**	25.27 ± 1.78	26.55 ± 1.60	26.58 ± 1.88	26.94 ± 0.38
**APTT (Sec)**	33.17 ± 4.50	25.06 ± 4.98^***^↓	28.76 ± 4.23	28.82 ± 3.27

All figure facts are in mean ± SD, *P<0.05, **P<0.01 and ***P<0.001 when compared to VC. ↓-Decrease, ↑-Increase.

**Table 5:** Results of 90 days repeated dose administration of (S)-Equol on total blood count in female Sprague Dawley rats.

**Hematology - Females**				
**Parameters**	**Vehicle control**	**250 mg/kg/day**	**500 mg/kg/day**	**100 mg/kg/day**
**WBC (10**^**3**^**cells/µL)**	7.94 ± 2.89	7.50 ± 1.87	5.91 ± 1.55	5.99 ± 1.78
**RBC (10**^**6**^**cells/µL)**	7.90 ± 0.52	8.14 ± 0.28	7.51 ± 0.47	7.46 ± 0.46
**HGB (g/dL)**	14.44 ± 0.52	14.64 ± 0.30	13.66 ± 0.68^**^↓	13.55 ± 0.65^**^↓
**HCT (%)**	48.48 ± 2.63	49.46 ± 1.52	45.59 ± 2.69*↓	45.85 ± 2.14*↓
**MCV (fL)**	61.45 ± 1.49	60.83 ± 2.10	60.72 ± 0.93	61.50 ± 1.75
**MCH (pg)**	18.32 ± 0.68	18.00 ± 0.49	18.19 ± 0.52	18.17 ± 0.49
**MCHC (g/dL)**	29.82 ± 0.61	29.60 ± 0.53	29.95 ± 0.50	29.54 ± 0.31
**Neu (10**^**3**^**cells/µL)**	0.86 ± 0.31	1.25 ± 1.22	0.76 ± 0.38	0.97 ± 0.57
**Lymph (10**^**3**^**cells/µL)**	6.54 ± 2.68	5.90 ± 1.27	4.90 ± 1.31	4.74 ± 1.21
**Mono (10**^**3**^**cells/µL)**	0.25 ± 0.10	0.22 ± 0.11	0.15 ± 0.04	0.15 ± 0.07
**Eos (10**^**3**^**cells/µL)**	0.22 ± 0.20	0.09 ± 0.04*↓	0.06 ± 0.02^**^↓	0.09 ± 0.04*↓
**Baso (10**^**3**^**cells/µL)**	0.01 ± 0.01	0.01 ± 0.01	0.01 ± 0.01	0.00 ± 0.01
**Retic (10**^**3**^**cells/µL)**	152.90 ± 40.45	154.56 ± 66.03	129.06 ± 25.06	137.02 ± 26.03
**Platelet (10**^**3**^**cells/µL)**	798.50 ± 121.14	864.30 ± 109.78	898.90 ± 112.59	999.70 ± 127.32^**^↑
**PT (Sec)**	26.34 ± 2.04	31.53 ± 2.11^***^↑	30.31 ± 2.83^**^↑	31.10 ± 2.24^***^↑
**APTT (Sec)**	29.22 ± 1.77	33.75 ± 3.05^**^↑	35.63 ± 3.32^***^↑	31.46 ± 4.25

All figure facts are in mean ± SD, *P<0.05, **P<0.01 and ***P<0.001 when compared to VC. ↓-Decrease, ↑-Increase.

**Table 6:** Results of 90 days repeated dose administration of (S)-Equol on clinical chemistry in male Sprague Dawley rats.

**Clinical Chemistry - Males**				
**Parameters**	**Vehicle control**	**250 mg/kg/day**	**500 mg/kg/day**	**100 mg/kg/day**
**GLUC (mg/dL)**	133.50 ± 8.42	125.10 ± 6.9	127.00 ± 13.53	122.70 ± 11.02
**BUN (mg/dL)**	12.20 ± 1.75	14.50 ± 4.12	14.90 ± 3.35	13.00 ± 3.20
**CREA (mg/dL)**	0.25 ± 0.07	0.29 ± 0.06	0.30 ± 0.06	0.33 ± 0.06*↑
**CHOL (mg/dL)**	64.70 ± 9.94	19.90 ± 4.77^***^↓	21.40 ± 4.53^***^↓	25.00 ± 8.06^***^↓
**TGL (mg/dL)**	48.40 ± 17.93	106.50 ± 43.15^**^↑	93.70 ± 44.73*↑	74.20 ± 26.33
**TP (g/dL)**	7.13 ± 0.29	7.22 ± 0.27	7.30 ± 0.23	7.54 ± 0.33^**^↑
**ALB (g/dL)**	1.14 ± 0.12	1.16 ± 0.07	1.28 ± 0.06^**^↑	1.27 ± 0.08^**^↑
**TBI (mg/dL)**	0.15 ± 0.10	0.10 ± 0.00	0.10 ± 0.07	0.10 ± 0.05
**AST (U/L)**	78.80 ± 15.65	63.50 ± 8.51*↓	64.80 ± 5.45	57.00 ± 12.30^***^↓
**CA (mg/dL)**	9.57 ± 0.26	9.29 ± 0.32	8.66 ± 0.38^***^↓	8.28 ± 0.36^***^↓
**PHOS (mg/dL)**	5.82 ± 0.50	5.66 ± 0.42	5.79 ± 0.32	5.50 ± 0.35
**ALP (U/L)**	80.90 ± 17.70	148.40 ± 34.41^***^↑	171.60 ± 12.32^***^↑	173.60 ± 45.51^***^↑
**HDL (mg/dL)**	56.10 ± 8.49	13.90 ± 4.01^***^↓	15.70 ± 4.99^***^↓	16.30 ± 5.70^***^↓
**LDL (mg/dL)**	10.60 ± 2.12	3.90 ± 1.20^***^↓	4.30 ± 1.95^***^↓	6.60 ± 4.55^**^↓
**ALT (U/L)**	49.20 ± 10.62	47.90 ± 9.87	54.00 ± 9.44	53.10 ± 19.66
**GGT (U/L)**	6.60 ± 1.26	7.60 ± 1.07	7.30 ± 0.82	8.80 ± 1.81^**^↑
**Na^+^ (mmol/L)**	137.40 ± 1.17	137.04 ± 0.76	137.52 ± 0.64	137.62 ± 0.81
**K^+^ (mmol/L)**	4.21 ± 0.28	4.266 ± 0.42	4.24 ± 0.41	3.86 ± 0.25
**Cl^-^ (mmol/L)**	102.66 ± 0.79	107.15 ± 11.62	103.27 ± 1.34	102.51 ± 0.95
**GLOB (calc) (g/dL)**	5.99 ± 0.25	6.06 ± 0.29	6.02 ± 0.22	6.27 ± 0.35
**TSH (ng/mL)**	4.0 ± 3.2	2.8 ± 1.7	2.9 ± 0.6	3.6 ± 2.0
**T4 (ng/mL)**	210.1 ± 58.7	208.2 ± 48.8	209.7 ± 44.9	182.8 ± 28.7
**T3 (ng/mL)**	9.2 ± 0.8	9.9 ± 1.3	9.3 ± 1.1	11.3 ± 1.9*↑

All figure facts are in mean ± SD, *P<0.05, **P<0.01 and ***P<0.001 when compared to VC. ↓-Decrease, ↑-Increase.

**Table 7:** Results of 90 days repeated dose administration of (S)-Equol on clinical chemistry in female Sprague Dawley rats.

**Clinical Chemistry - Females**				
**Parameters**	**Vehicle control**	**250 mg/kg/day**	**500 mg/kg/day**	**100 mg/kg/day**
**GLUC (mg/dL)**	136.20 ± 11.89	136.20 ± 11.65	128.60 ± 7.57	132.00 ± 10.17
**BUN (mg/dL)**	15.90 ± 2.56	17.50 ± 4.53	17.40 ± 3.75	22.40 ± 4.20^**^↑
**CREA (mg/dL)**	0.33 ± 0.07	0.35 ± 0.08	0.37 ± 0.04	0.36 ± 0.08
**CHOL (mg/dL)**	78.60 ± 14.65	28.80 ± 8.90^***^↓	33.20 ± 12.42^***^↓	34.40 ± 7.63^***^↓
**TGL (mg/dL)**	54.70 ± 21.28	89.90 ± 58.74	82.70 ± 30.79	75.30 ± 29.22
**TP (g/dL)**	8.02 ± 0.57	8.28 ± 0.45	8.53 ± 0.60	8.30 ± 0.71
**ALB (g/dL)**	1.60 ± 0.24	1.44 ± 0.16	1.49 ± 0.23	1.47 ± 0.25
**TBI (mg/dL)**	0.08 ± 0.04	0.12 ± 0.04	0.10 ± 0.05	0.12 ± 0.04
**AST (U/L)**	92.80 ± 24.48	66.00 ± 10.81^***^↓	54.50 ± 5.06^***^↓	48.40 ± 7.86^***^↓
**CA (mg/dL)**	9.63 ± 0.37	8.63 ± 0.31^***^↓	8.90 ± 0.48^**^↓	8.29 ± 0.68^***^↓
**PHOS (mg/dL)**	5.27 ± 0.59	4.79 ± 0.38	4.73 ± 0.43	5.21 ± 0.50
**ALP (U/L)**	54.10 ± 18.41	103.40 ± 30.43	92.20 ± 26.68	187.30 ± 77.54^***^↑
**HDL (mg/dL)**	66.30 ± 12.84	12.10 ± 4.31^***^↓	12.40 ± 1.96^***^↓	12.30 ± 3.43^***^↓
**LDL (mg/dL)**	6.90 ± 1.45	8.40 ± 4.58	10.50 ± 6.20	12.80 ± 4.21*↑
**ALT (U/L)**	49.10 ± 13.12	42.60 ± 8.63	37.60 ± 5.36	53.30 ± 14.82
**GGT (U/L)**	6.60 ± 1.07	8.20 ± 1.75	10.30 ± 2.11^***^↑	12.00 ± 2.26^***^↑
**Na^+^ (mmol/L)**	135.72 ± 1.33	135.99 ± 1.24	136.39 ± 0.69	136.64 ± 1.26
**K^+^ (mmol/L)**	3.877 ± 0.31	3.90 ± 0.29	3.88 ± 0.22	4.167 ± 0.57
**Cl^-^ (mmol/L)**	103.33 ± 0.68	101.54 ± 1.88*↓	102.42 ± 1.06	102.72 ± 1.84
**GLOB (calc) (g/dL)**	6.42 ± 0.35	6.84 ± 0.39	7.04 ± 0.43^**^↑	6.83 ± 0.50
**TSH (ng/mL)**	1.9 ± 0.2	3.0 ± 2.1	2.3 ± 0.6	2.9 ± 0.8
**T4 (ng/mL)**	126.5 ± 29.3	167.6 ± 57.6	154.7 ± 88.4	140.9 ± 36.0
**T3 (ng/mL)**	6.9 ± 1.5	7.3 ± 1.4	7.9 ± 1.6	7.7 ± 1.4

All figure facts are in mean ± SD, *P<0.05, **P<0.01 and ***P<0.001 when compared to VC. ↓-Decrease, ↑-Increase.

The animals treated with high dose (1000 mg/kg) of test item showed decreased WBC, RBC, lymphocyte count in males and haemoglobin and haematocrit in both sexes when compared to concurrent vehicle control. Furthermore, absolute eosinophils were found to decrease in high dose females. However, platelet count increased in both sexes of high dose group. In every test item treated female, prothrombin time and activated partial thromboplastin clotting time have increased.

#### 3.3.8 Hormones

The study found TSH, T4, and T3 parameters in both sexes comparable to control animals, but T3 levels increased significantly in high dose males. The observed T3 levels were with in historical control data of laboratory and there was no correlated microscopic lesions observed in thyroid and parathyroid, hence considered as a non-adverse Table 6, Table 7.

#### 3.3.9 Pathology

##### 3.3.9.1 Gross necropsy

In any of the animals, at any tested dose level, or in a concurrent vehicle control, no discernible pathological alterations were seen.

##### 3.3.9.2 Organ weights

Repeated dose administration of a 90-day dose altered organ weights, resulting in a compound-related increase in relative adrenal weight in all treated groups. This increase was more noticeable in G4 males and was correlated with histopathological evaluation in all (S)-Equol treated groups. On day 91, fasted body weight was lesser due to less body weight gain in the study, this could be responsible for significance in relative organ weight observed. The relative brain weight to organ weight was not significance hence, considered as non-adverse effect Table 8, Table 9, Table 10, Table 11.

**Table 8:** Effect of 90 days repeated dose administration of (S)-Equol on organ weight in male rats.

Absolute Organ Weight - Males				
Parameters	Vehicle control	250 mg/kg/day	500 mg/kg/day	100 mg/kg/day
Liver (g)	15.9763 ± 2.7629	15.6033 ± 2.6169	16.4473 ± 2.9368	16.0529 ± 3.0263
Kidneys (g)	3.6307 ± 0.3578	3.3598 ± 0.4990	3.5453 ± 0.7747	3.0388 ± 0.5157
Heart (g)	1.9004 ± 0.2249	1.5621 ± 0.2240^**^↓	1.5051 ± 0.1561^***^↓	1.5405 ± 0.2591^**^↓
Adrenals (g)	0.0636 ± 0.0071	0.0716 ± 0.0104	0.0740 ± 0.0143	0.0904 ± 0.0131^***^
Thymus (g)	0.4452 ± 0.0842	0.3752 ± 0.0907	0.3809 ± 0.1067	0.348 ± 0.0988
Spleen (g)	0.8345 ± 0.1023	0.7631 ± 0.1540	0.7464 ± 0.1466	0.7002 ± 0.1999
Brain (g)	2.2371 ± 0.0773	2.1946 ± 0.1046	2.1736 ± 0.1034	2.1639 ± 0.1024
Testes (g)	3.7692 ± 0.2973	3.4230 ± 0.3380	3.3115 ± 0.2207*↓	2.9925 ± 0.4341^***^↓+
Epididymides (g)	1.6360 ± 0.1076	1.376 ± 0.1754	1.3635 ± 0.1624	1.4788 ± 1.2170
PSVC (g)	3.8649 ± 0.6669	2.4794 ± 0.7118^***^↓	2.6118 ± 0.7743^**^↓	1.7556 ± 0.7685^***^↓
Pituitary (g)	0.0151 ± 0.0055	0.0136 ± 0.0056	0.0133 ± 0.0072	0.0155 ± 0.0032
Thyroid & Parathyroid (g)	0.0258 ± 0.0030	0.0227 ± 0.0049	0.0271 ± 0.0034	0.0252 ± 0.0035

All figure facts are in mean ± SD, *P<0.05, **P<0.01 and ***P<0.001 when compared to VC. ↓-Decrease, ↑-Increase.

**Table 9:** Results of 90 days repeated dose administration of (S)-Equol on organ weight in female rats.

**Absolute Organ Weight - Females**				
**Parameters**	**Vehicle control**	**250 mg/kg/day**	**500 mg/kg/day**	**100 mg/kg/day**
**Liver (g)**	10.1313 ± 1.4084	11.2932 ± 1.3356	10.1649 ± 0.7200	10.7511 ± 1.4343
**Kidneys (g)**	2.1556 ± 0.1889	2.1667 ± 0.2629	1.9002 ± 0.2015*↓	1.9673 ± 0.2189
**Heart (g)**	1.1901 ± 0.0610	1.2301 ± 0.2106	1.0466 ± 0.0825	1.0651 ± 0.1242
**Adrenals (g)**	0.0790 ± 0.02170	0.1007 ± 0.024	0.0981 ± 0.022	0.0930 ± 0.0141
**Thymus (g)**	0.3937 ± 0.1136	0.3558 ± 0.0679	0.2894 ± 0.0632*↓	0.2548 ± 0.0751^**^↓
**Spleen (g)**	0.6134 ± 0.1835	0.5718 ± 0.0469	0.5173 ± 0.0715	0.5120 ± 0.1149
**Brain (g)**	2.0898 ± 0.1098	2.1106 ± 0.0982	2.0659 ± 0.1000	2.0403 ± 0.1085
**Ovaries (g)**	0.1665 ± 0.0331	0.1587 ± 0.0399	0.1392 ± 0.0523	0.1410 ± 0.0320
**Uterus with cervix (g)**	0.9339 ± 0.3513	0.7726 ± 0.3044	0.7024 ± 0.1711	0.8081 ± 0.6009
**Pituitary (g)**	0.0184 ± 0.0046	0.0167 ± 0.0040	0.0151 ± 0.0066	0.0137 ± 0.0038*↓
**Thyroid & Parathyroid (g)**	0.0217 ± 0.0052	0.0218 ± 0.0057	0.0202 ± 0.0043	0.0199 ± 0.0025

All figure facts are in mean ± SD, *P<0.05, **P<0.01 and ***P<0.001 when compared to VC. ↓-Decrease, ↑-Increase.

**Table 10:** Results of 90 days repeated dose administration of (S)-Equol on relative brain weight to organ weight in male rats.

**Relative Brain weight to Organ Weight - Males**				
**Parameters**	**Vehicle control**	**250 mg/kg/day**	**500 mg/kg/day**	**100 mg/kg/day**
**Brain Weight (**%**)**	2.24 ± 0.08	2.19 ± 0.10	2.17 ± 0.10	2.16 ± 0.10
**Liver (%)**	715.19 ± 130.56	708.70 ± 100.20	755.59 ± 124.51	739.74 ± 119.39
**Kidneys (%)**	162.47 ± 17.43	152.76 ± 19.22	163.30 ± 36.39	140.23 ± 21.94
**Heart (%)**	84.99 ± 10.31	70.97 ± 8.03^**^↓	69.23 ± 6.19^**^↓	71.19 ± 11.35^**^↓
**Adrenals (%)**	2.85 ± 0.36	3.26 ± 0.42	3.42 ± 0.73	4.18 ± 0.60^***^↑
**Thymus (%)**	19.94 ± 4.01	17.10 ± 4.14	17.45 ± 4.59	16.13 ± 4.82
**Spleen (%)**	37.36 ± 4.94	34.72 ± 6.58	34.38 ± 6.87	32.18 ± 8.35
**Testes (%)**	168.87 ± 17.23	156.16 ±15.60	152.70 ± 13.14	137.89 ± 15.94
**Epididymides (%)**	73.23 ± 5.85	62.73 ± 7.87*↓	62.82 ± 7.74*↓	66.83 ± 50.90^***^↓
**PSVC (%)**	172.92 ± 31.04	112.27 ± 30.43^***^↓	120.84 ± 38.44^**^↓	80.57 ± 35.02^***^↓
**Pituitary (%)**	0.68 ± 0.25	0.62 ± 0.25	0.61 ± 0.33	0.71 ± 0.13
**Thyroid & Parathyroid (%)**	1.16 ± 0.17	1.03 ± 0.22	1.25 ± 0.18	1.17 ± 0.19

All figure facts are in mean ± SD, *P<0.05, **P<0.01 and ***P<0.001 when compared to VC. ↓-Decrease, ↑-Increase.

**Table 11:** Results of 90 days repeated dose administration of (S)-Equol on relative brain weight to organ weight in female rats.

**Relative Brain weight to Organ Weight - Females**				
**Parameters**	**Vehicle control**	**250 mg/kg/day**	**500 mg/kg/day**	**100 mg/kg/day**
**Brain Weight (**%**)**	2.09 ± 0.11	2.11 ± 0.10	2.07 ± 0.10	2.04 ± 0.11
**Liver (%)**	485.89 ± 74.87	535.27 ± 60.81	492.93 ± 41.83	526.21 ± 57.40
**Kidneys (%)**	103.45 ± 11.33	102.94 ± 14.21	92.02 ± 8.84	96.33 ± 8.39
**Heart (%)**	57.13 ± 58.25	58.25 ± 9.32	50.67 ± 3.13	52.17 ± 5.09
**Adrenals (%)**	3.81 ± 1.17	4.76 ± 1.08	4.74 ± 0.99	4.55 ± 0.63
**Thymus (%)**	18.97 ± 5.87	16.88 ± 3.29*↓	14.03 ± 3.11^**^↓	12.49 ± 3.58
**Spleen (%)**	29.42 ± 9.03	27.10 ± 1.95	25.05 ± 3.33	25.08 ± 5.27
**Ovaries (%)**	7.26 ± 1.52	7.48 ± 1.74	6.68 ± 2.30	6.92 ± 1.53
**Uterus with cervix (%)**	45.15 ± 17.93	36.79 ± 15.24	33.96 ± 7.71	39.54 ± 29.16
**Pituitary (%)**	0.88 ± 0.22	0.79 ± 0.20	0.73 ± 0.31	0.67 ± 0.17
**Thyroid & Parathyroid (%)**	1.04 ± 0.27	1.04 ± 0.29	0.97 ± 0.18	0.98 ± 0.15

All figure facts are in mean ± SD, *P<0.05 and **P<0.01 when compared to VC. ↓-Decrease.

##### 3.3.9.3 Histopathological finding

**Fig. 2:**
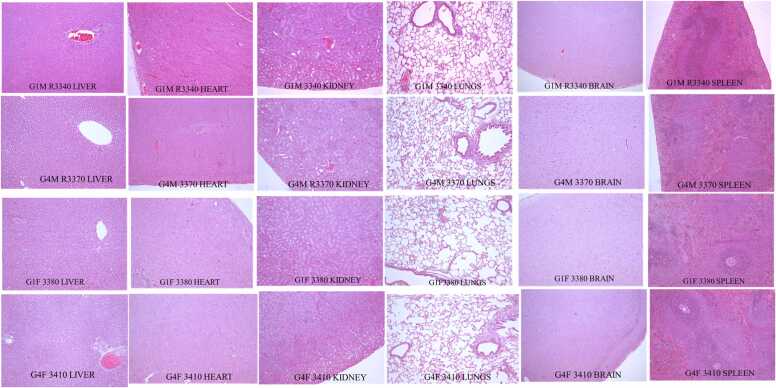
**: Representative microscopic images of organs from sub-chronic study.** G1M & G1F - Vehicle control male and female respectively; G4M & G4F - 1000 mg/kg/day (S)-Equol treated males and females respectively.

Microscopic examination revealed compound-related cortical hypertrophy in adrenals of all test item treated groups, with highest incidence in high dose males and females. Zona fasciculata of adrenal cortex cells were mostly hypertrophied, with increased size and eosinophilic homogenous cytoplasm, compared to vehicle control animals with no cytoplasmic vacuolation. The all other major organs showed no microscopic lisions. The representative microscopic lisions updated from vehicle controle and high dose treated droup animals (Fig. 2).

## 4 Discussion

The oral toxicity acute study showed that (S)-Equol was tolerable up to 2000 mg/kg in female Sprague Dawley rats and (S)-Equol was classified into GHS category 5/Unclassified. When (S)-Equol was given orally, every day for 14 days consecutively, the NOAEL in both the sexes were found to be 160 mg/kg/day. Reduction of total cholesterol and triglycerides observed was considered as a pharmacological effect of (S)-Equol. The general human consumption dose of (S)-Equol is 10–40 mg/day. With the maximal human dose of 40 mg/day (0.66 mg/kg) as a food, supplement, the highest dose 160 mg/kg/day in rats was 242 folds of human dose and showed no adverse effect on the parameters evaluated.

The test dose levels for subchronic study was 250, 500 and 1000 mg/kg body weight. These dose levels were 379, 758 and 1515 fold higher considering the human dose respectively. Reduction in body weight was observed compared to vehicle control. However, when the body weights of day 1 was compared to day 90, animals have gained the body weight. The gain in body weight and feed consumption was not comparable to vehicle control group. The reduced feed consumption is correlated with decrease in body mass gain. The rapid body weight gain for Sprague Dawley rats were usually observed at 5–8 weeks age and decreases further. and decreased further. The sub-chronic study dosing was started at age ∼ 5 weeks’ animals. For a considerable weight gain, a protein synthesis is required which uses the cholesterol in the body. With the treatment of (S)-Equol, a significance reduction in total cholesterol and the triglycerides could be responsible for reduced body weight gain. Estrogen usage has been shown to reduce BW gain and visceral fat development in both animals and humans. Furthermore, LeeCole L. and group, reported decrease in cholesterol levels by compound of similar class in ovariectomized rats [10]. The decrease in cholesterol level is considered as pharmacodynamics effect of the (S)-Equol [6].However, increased ALP, GGT without histoapthological correlation was observed. Hence, these observations considered as non-adverse.

All other parameters like total blood count, clinical chemistry, functional observation battery test, vaginal cytology, ophthalmology, and urine parameters showed no adverse effects. The oral administration of (S)-Equol upto 1000 mg/kg body weight (>1515 folds higher human dose) per day till 90 days did not show gross necropsy changes and also there is no microscopic changes observed for any of the organs except adrenals. Hence, NOAEL for female animals was 250 mg/kg body weight. The NOAEL for male rats was below 250 mg/kg/day. The percent reduction in body weight gain was considered as a pharmacology class of effect and further investigation required at low doses and recovery group of animals. However, the LOAEL for male rats was 250 mg/kg/day considered. Grounded on the outcomes of the present repeated-dose toxicity, the use of (S)-Equol as dietary supplement in humans is anticipated to be safe.

This increased adrenal weight was considered to be prolong stress induced due to continuous decreased body weight gain in all the treated groups specially in males as compared to females. These findings are in accordance with the literature [2], [24] who reported adrenal cortex response to prolonged stress or ACTH administration. Similar findings in adrenal was reported by Mazzocchi and group [15]. On prolong administration of 4-aminopyrazolo-pyrimidine which, reduces the serum cholesterol level in rat. Belloni et. al. also reported similar findings in zona fasciculate of adrenal cortex on administration of mevinolin (lovastatin) in rats [1]. This changes correlated with decreased cholesterol levels observed in the (S)-Equol treated animals. This observed histopathological change in adrenals was considered as non-adverse as no changes in effect on the behaviour or functionality of the animals were observed during the experimental period. This consideration was supported by Preter Greaves who reported no problems in clinical practice on use of similar changes induced in animals by compound lovastatin [1]. All these changes even though reached the statistical significance are within the historical control range hence considered toxicologically non-significant except PT and APTT in females, besides, no histopathological correlation was observed on microscopic evaluation. Hence increased PT and APTT in females is considered as non-adverse. Since the other findings in all of the study animals were inconsistent and of a sporadic nature, they were regarded as incidental or background to the Sprague Dawley rats that were employed in the investigation. (S)-Equol was well tolerated by postmenopausal women in 14 days repeated dose BID oral administration of 10, 20, 40, 80, and 160 mg. No drug-related contrary effects were found even at highest dose tested till 14 days of administration. (S)-Equol was well tolerated by postmenopausal women confirms the safety of (S)-Equol [8]. Since the consumption of (S)-Equol was majorly in menopausal condition and at old age. The consumption of (S)-Equol will not have any health problem.

The toxico-kinetics study of (S)-Equol in rat and monkey demonstrated safety at 25, 125 and 500 mg/kg b.w. and the induction of Cyp P450 was minimal [21]. The consumption of soya rich diet up to 2000 mg/kg/day (equivalent to 13 mg equol/kg/day) and 1000 mg/kg/day (equivalent to 6.5 mg equol/kg/day) in female rats was NOAEL for developmental and reproductive toxicity study respectively [13]. The soy rich product containing Equol from fermentation process showed the NOAEL of 2000 mg/kg/day and LD_50_ of >4000 mg/kg/day in rats and also reported negative for *in vitro* chromosomal aberrations and *in vivo* clastogenic effect [29].

## 5 Conclusions

The study reports valuable insights into the safety and pharmacological effects of (S)-Equol. The acute toxicity study showed that (S)-Equol is safe at > 2000–5000 mg/kg body weight and it classified into GHS category 5/Unclassified for classification of chemicals and mixtures. In the subacute or dose range finding study, the NOAEL was found to be 160 mg/kg body weight with a as safety factor of 242 times of human dose which showed no adverse drug effect in both gender. The sub-chronic study revealed a substantial safety factor of (S)-Equol compared to highest human dose. Despite a reduction in body weight gain correlated with decreased feed consumption, other health parameters, including total blood count, clinical chemistry, and organ weights and on histopathological evaluation of tissues, showed no adverse effects. Specifically, NOAEL for female rats was 250 mg/kg/day and LOAEL for male rats was 250 mg/kg/day.

## CRediT authorship contribution statement

**Ananda Kumar S R:** Writing – review & editing, Writing – original draft, Software, Methodology, Investigation, Formal analysis, Data curation. **Mukund Handral:** Methodology, Investigation. **Srinivas Seekallu:** Writing – review & editing, Methodology, Investigation.

## Declaration of Competing Interest

The authors declare that they have no known competing financial interests or personal relationships that could have appeared to influence the work reported in this paper.

## Data Availability

Data will be made available on request.
